# Cystocele Repair by a Modified Surgical Technique of Bilateral Pubococcygeus Plication: Long-Term Surgical and Functional Results

**DOI:** 10.3390/jcm9103318

**Published:** 2020-10-16

**Authors:** Matteo Balzarro, Emanuele Rubilotta, Alessandro Antonelli

**Affiliations:** Department of Urology, Azienda Ospedaliera Universitaria Integrata Verona, Piazzale Stefani 1, 37100 Verona, Italy; emanuele.rubilotta@aovr.veneto.it (E.R.); alessandro_antonelli@me.com (A.A.)

**Keywords:** cystocele repair, anterior vaginal wall defect repair, native tissue repair

## Abstract

Here we describe our modified surgical technique of bilateral pubococcygeus plication (BPCP) for cystocele repair and assess its safety and long-term outcomes. This is a prospective study of 147 consecutive women who underwent BPCP for cystocele between January 2010 to January 2018. Inclusion criteria was naïve women with symptomatic cystocele ≥ POP-Q 2nd stage. Exclusion criteria: stress urinary incontinence (SUI), urgency urinary incontinence, other associated vaginal wall prolapses ≥ stage 2, neurological diseases, previous SUI surgeries, and previous radiation/surgery of the pelvic area. BPCP was performed by obtaining the medialization of the pubococcygeus muscle fibers of the right and left sides. Statistical analysis was performed. Objective cure was POP-Q < 2nd stage. Subjective cure and functional outcomes were evaluated by validated questionnaires. Patient’s satisfaction was assessed by a Likert-type scale. Mean operative time was 64 min. At a mean follow-up of 82.4 months, objective and subjective success rates were 89.8% and 92.2% respectively. De novo urgency was 3.2%. Surgery did not alter sexual function. Complications occurred in 4.8%, and were: wrong dissection plane, hematoma, and pain lasting between 24–72 h. BPCP for correction of cystocele is safe and effective, with limited risk of complication and good long-term results.

## 1. Introduction

Pelvic organ prolapse (POP) is a common pathological condition affecting millions of women, and its incidence is likely to increase further because of aging populations [1].

Concerning the surgical treatment of the anterior compartment, several materials including native tissue, allograft, xenograft, and mesh have been used. In the last decade, the large use of trans-vaginal mesh for anterior vaginal wall repair to ensure greater effectiveness and duration of treatment has greatly reduced the use of native tissue surgical techniques. Nevertheless, surgeons who prefer the use of native tissue for anterior vaginal repair researched modified techniques to improve the outcomes. The high rate of mesh-related complications became an issue and led many countries to suspend their use. To date, data on surgical treatments with native, or autologous, tissues that guarantee duration and efficacy of results is a priority.

The high rate of mesh complications, and the poor outcomes in the long term with anterior colporrhaphy, lead us to research a stronger native support for anterior vaginal wall repair. We found this native reinforcement in the modified bilateral pubococcygeus plication (BPCP). The aim of this study is to describe and illustrate BPCP, and to report the long-term outcomes in treating patients with cystocele in a tertiary referral setting.

## 2. Experimental Section

### 2.1. Data Acquisition

Data were prospectively collected from women undergoing BPCP for the treatment of anterior vaginal wall defect from January 2010 to January 2018. Inclusion criteria was naïve women with symptomatic cystocele > POP-Q 2nd stage. The research procedures were performed according to the Declaration of Helsinki. Patients gave their informed and written consent to the study. The Ethics Committee did not consider it necessary to be involved as the clinical routine was not changed. However, this study was recorded within the department audit.

The research procedures were performed according to the Declaration of Helsinki. Patients gave their informed and written consent to the study. We enrolled only women with strict criteria to avoid bias, and only some women were suitable for the study.

Data collected consisted of: (i) Demographic details; (ii) Objective anterior vaginal wall defect measurement by Pelvic Organ Prolapse Quantification (POP-Q) system assessed by maximum Valsalva effort in the seated semi-lithotomy position; (iii) Subjective evaluation by the following validated questionnaires: the Patient Global Impression of Improvement (PGI-I) and Patient Perception of Bladder Condition (PPBC) [2,3]; (iv) Female sexual function by Female Sexual Function Index (FSFI) questionnaire [4]; (v) OAB-screener questionnaire.

Urodynamic evaluation was in the study design to ensure an accurate functional evaluation, and to uncover occult SUI. Urodynamics included a first study without POP reduction, and a second study with POP reduction by pessary placement. Comprehensive pre-operative counseling and shared decision-making framework were adopted to decide on the technique for anterior vaginal wall prolapse management [5]. Exclusion criteria for this study were: stress urinary incontinence (SUI), urgency urinary incontinence, other associated vaginal wall prolapses ≥ stage 2, neurological diseases, previous SUI surgeries, and previous radiation/surgery of the pelvic area.

Surgery was performed by two surgeons (M.B., E.R.) using a standardized technique (see Section 2.2). Any intra- or postoperative complications, as well as any further surgical treatments undertaken, were prospectively recorded in a form by a third physician not involved in the procedure. The Clavien–Dindo classification was used to rank complications. Patients were reviewed in the outpatient clinic at 6 weeks, and an assessment of symptoms was undertaken along with vaginal examination to ensure healing had taken place. Women were routinely followed by an examiner not directly involved in the surgical procedure at an annual scheduled visit to evaluate objective and subjective results, and female sexual function.

Outcomes were assessed by maximum Valsalva effort in the seated semi-lithotomy position. Objective cure was defined as when the anterior vaginal wall was inferior to the POP-Q 2nd stage, while any anterior vaginal wall defects ≥2nd stage were considered failures. Subjective cure rates were evaluated by PGI-I and PPBC questionnaires considering satisfied patients with PGI-I ≤ 3, and PPBC ≤ 2. Sexual impact of the surgical procedure was assessed through post-operative FSFI questionnaires. De novo storage symptoms were assessed by direct questioning and voiding diaries for 3 days. Before assessing de novo storage symptoms, all patients had to have negative urine culture. Urinary retention was ascertained by postvoid clean catheterization postoperatively and at the clinic follow-up. Outcomes of these data have been statistically evaluated by T-Student test.

Finally, to better investigate patient’s personal satisfaction, we asked the following questions by a Likert-type scale: (i) “Are you satisfied with the surgical procedure?” (ii) “Would you confirm the same surgical choice during the counseling before surgery?”

### 2.2. Surgical Technique

**Figure 1:**
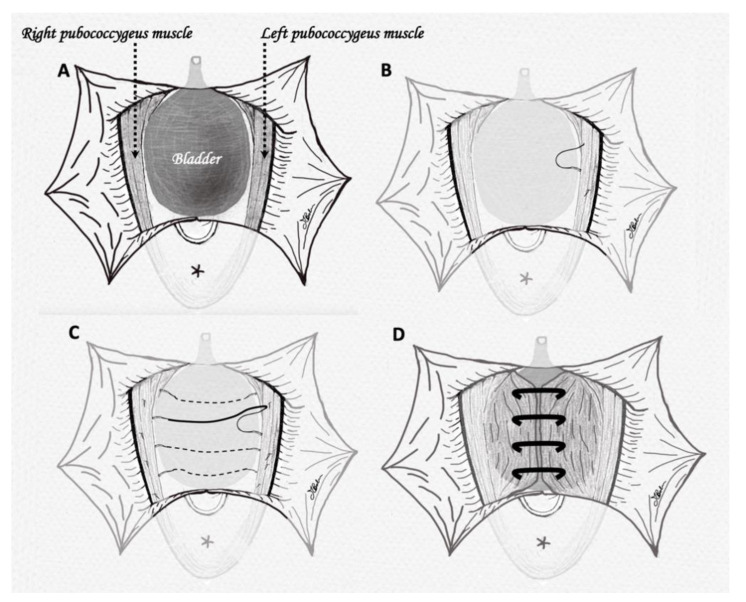
(**A**) Large dissection of bladder and exposure of pubococcygeus muscle fibers; (**B**) placement of the first suture on left side fibers; (**C**) all 4 sutures have been placed on both sides of pubococcygeus muscles; (**D**) sutures have been tightened, obtaining plication of the pubococcygeus fibers.

The patient was given general or spinal anesthesia and broad-spectrum antibiotic prophylaxis and then placed in the lithotomy position, with indwelling catheter. A Scott retractor was placed with 6 hooks symmetrically positioned in the vagina to separate the labia and facilitate vaginal dissection. A 3–4 cm midline incision was made through the vaginal wall at 0.5–1 cm below the bladder neck, located by pulling on the Foley catheter and palpating the balloon. Two Allis forceps were placed through the lateral edge of the wound, providing tension to facilitate the dissection of vaginal wall off the underlying pubocervical fascia until the prolapsed bladder was completely dissected. The dissection was continued laterally in the direction of the obturator foramen, and posteriorly to the pubic arch until the fibers of the pubococcygeus muscle were exposed or distinctly palpable. The vaginal dissection was laterally extended. The placement of a gauze into the dissected area gently pushing the bladder into its normal position facilitated the dissection. Vicryl CT-2 plus 0 sutures were placed laterally on the pubococcygeus muscle, avoiding passing through the vaginal wall. Sutures went from top to bottom, in order to ease the maneuver, and avoid potential injury of the terminal ureter. In this step, the surgeon should not be afraid of having taken too much tissue. Each suture remained unknotted with the needle in mosquito forceps, so that the correct placement could be checked by pulling the suture and feeling strong tissue resistance in traction. When 3–4 sutures were placed in the left side, the procedure was repeated contralaterally, ideally hanging the pubococcygeus muscle at the same level to create symmetric tension. Suture tensioning aimed to medialize the pubococcygeus muscle fibers of the right and left sides, ensuring an adequate vaginal caliber and avoiding narrowing with consequent dyspareunia. Pulled sutures were then tied. Figure 1 shows the crucial passages of our technique. To prevent excessive tightness of the anterior vaginal wall we suggest air knots instead of tight sutures. Finally, the vaginal wall was trimmed from the flaps bilaterally, and standard anterior colporrhaphy and anterior vaginal wall closure were performed. We inserted a vaginal pack that could be removed with the catheter after 48 h. Finally, wound dressing was carried out once a day with the use of a vaginal soft-gel capsule containing silver nanoparticles hydrogel (Tiagin Fast^®^) for 14 days. Sport, sexual activity, or any other strenuous activity were avoided by patients for 4–6 weeks.

## 3. Results

### 3.1. Demographics and Indication for Surgery

**Figure 2:**
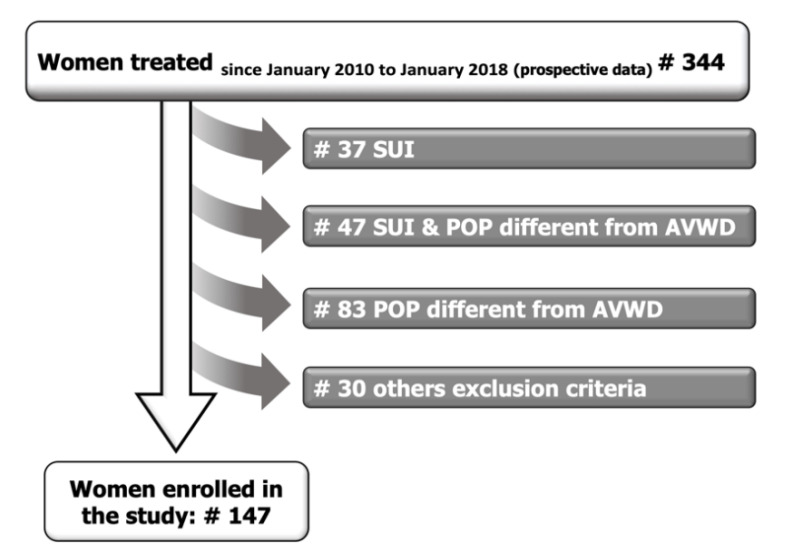
Study design diagram.

A total of 344 patients were treated. However, only 147 naïve women were suitable for this study (Figure 2. The mean age was 66.8 (SD 10) yr. POP-Q stage and mean Aa/Ba are reported in Table 1. Patients with a uterus descent below stage 2 POP-Q all had an anterior vaginal wall defect stage 3 (mean C point-5). In all of these patients, the uterine defect did not worsen, but improved after the surgery. All selected women had no occult stress urinary incontinence. Overactive bladder (OAB) was reported by 36.8%, but none had urinary incontinence.

### 3.2. Preoperative Functional and Urodynamic Characteristics

All individuals were symptomatic, reporting a feeling of pressure or fullness/bulging in the pelvic area (100%), feeling that something was falling out of the vagina (43%), painful intercourse (29.4%), obstructed voiding (25%), and spotting or bleeding from the vagina (5.2%). No patients demonstrated urodynamic SUI, while 41 patients demonstrated detrusor overactivity. Mean bladder capacity was 410 mL, and voiding dysfunctions were detected in 15% without POP reduction, which decreased to 4% after POP reduction.

### 3.3. Outcomes

The mean follow-up was 82.4 months (range 24–120). Mean operative time was 64 min (SD 20). Mean blood loss was 60 mL, and only one woman required blood transfusion. Median length of hospital stay was 2 days (range 1–5). A return to normal activities was within 2 weeks with the recommendation not to perform intense exercises within 6–7 weeks of surgery. Objective success rate was 89.8% (132/147). In total, 15 women had an anterior vaginal wall recurrence (10.2%). Table 2 shows data on objective success, and the recurrences are compared between the preoperative and follow-up POP-Q stages.

Subjective satisfaction rate at validated questionnaires was 92.2%; mean PGI-I was 1.9 (SD 1.2), while mean PPBC was 1.2 (SD 1.0). The Likert-type scales showed that 90% of women reported personal satisfaction, and 92% would confirm the same surgical choice. After surgery, 9.8% of women reporting a feeling of pressure or fullness/bulging in the pelvic area, with the feeling that something was falling out of the vagina reported by 2 patients (1.4%).

Preoperative OAB rate was 36.7% (54/147), while after surgery this rate significantly decreased by 61.1% (33/54). Postoperative storage symptoms were observed in 5.4% (5/93), but in only 3.2% (3/93) of patients, these symptoms occurred de novo. OAB-screener score significantly improved at follow-up. Table 3 reports data on OAB symptoms. None of the patients had post void residuals, and recurrent urinary tract infections occurred in five (3.4%) patients. Pain was not reported as a relevant feature in any patients at 4-weeks follow-up.

A total of 76 women were sexually active (51.7%). In this population, the surgery did not adversely affect sexual function, and all single domains significantly improved after surgical treatment (Table 4). Re-operation rate was 1.4% (2/147); in one case a mesh was placed, while in the other the same technique was successfully performed.

### 3.4. Complications

We found that 4.8% of patients (7/147) had complications (Table 5): 2 intraoperative detrusor injuries due to a wrong dissection plane, intraoperatively successfully managed by suturing the wrong dissection; 3 cases of hematoma, one in a patient with a coagulation disorder conservatively managed by infusion of coagulation factors and platelets, and 2 requiring surgical intervention of hematoma drainage (Clavien–Dindo grade III); 2 patients with pain lasting between 24 and 72 h after the procedure managed with medication.

## 4. Discussion

To date there is no consensus on the best treatment for cystocele repair. The oldest native tissue repair technique is anterior colporrhaphy, which, since its first description in 1913, has undergone several modifications. Currently, most surgeons reconstruct the fibromuscular layer and adventitial layer to reapproximate both sides of the defect. Limits on anterior colporrhaphy data include low sample sizes, the anterior colporrhaphy definition between authors, and/or the short follow-up [6,7,8,9,10,11,12]. Furthermore, most of the retrospective studies did not use the POP evaluation suggested by the IUGA/ICS Joint Report on the Terminology for Female Pelvic Floor Dysfunction [13]. Thus, the parameters used to report the success rate are not homogeneous and poorly comparable [14,15]. Few RCTs report data on colporrhaphy [10,11,12,14]. Three studies presented the largest series, but with a follow-up lower than 2 years, and a success rate ranging from 37% to 84% [9,10,11]. Only two RCTs have long-term outcomes, but with very low sample sizes and mixed results [12,14]. Colombo et al. reported a success rate of 97% in 32 women, but additional associated procedures may have been a confounding variable [14]. Conversely, the last and most recent RCT series showed an anatomical success rate of 33% at 5–8 years in only 36 women [12].

Biologic and synthetic mesh have been introduced to improve surgical success rates of native tissue anterior repair. Comparative studies evaluated the use of prosthetic material compared to native tissue repair. Mesh placement had no statistically significant objective higher success rate. Furthermore, the use of prosthetic material was affected by a significantly higher complication rate [6,8,9,12]. The use of mesh for transvaginal prolapse repair has been strongly criticized in most anglophone countries. The recent FDA warning has almost excluded the use of trans vaginal mesh in the US market [16].

Finally, slings for anterior vaginal wall repair associate the vaginal and the abdominal routes. These procedures showed a success rate ranging from 43% to 100%. However, the abdominal surgical step may involve greater comorbidity and longer operating time [17,18]. Moreover, some of these techniques use a rectus fascia graft, increasing the invasiveness of the procedure [18].

Our experience on non-prosthetic anterior vaginal wall repair lead us to develop a modified surgical technique based on the use of a native tissue reinforcement to the standard anterior colporrhaphy. The plication of bilateral pubococcygeus fibers guarantees a stronger support as a substitute of mesh. Paparella et al. described the approximation of pubococcygeus muscles in patients with severe cystocele [19]. Compared to our technique, these authors used a more invasive and larger dissection, non-absorbable sutures to approximate pubococcygeus fibers, and plication under the bladder neck. The extension of the dissection to the bladder neck, and its involvement in the plication, probably caused the reported high rate of urinary retention (7%).

Our technique demonstrates a high objective and subjective cure rate at long-term follow-up, avoiding the use of synthetic material or autologous tissues. The main recurrences were low grade, and, of these, only a few required re-intervention. The BPCP is a fast and safe surgical option with no statistically significant negative impact on functional outcomes or sexual function. The use of silver nanoparticles hydrogels has been reported as a good wound dressing due to the antibacterial activity and tissue regeneration [20,21]. In the vagina, where it is not possible to do a wound dressing, the use of vaginal capsules of silver nanoparticles hydrogels has proved practical and effective in preventing vaginal discharge, wound healing complications, or vaginosis.

The positive objective and subjective success rates of our surgical technique are higher when compared with the RCTs previously reporting colporrhaphy [10,11,12,13,14]. BPCP provides a long-lasting support to colporrhaphy, making our modified surgical technique effective. A strength of our study is that outcomes were evaluated and analyzed by standardized methods. Confounding variables may have been reduced by the strict inclusion criteria of naïve women with only anterior vaginal wall defects and no other associated surgical repair. Our outcomes are strengthened by the large sample size and long-term follow-up. These two features together are missing in the reported RCTs [10,11,12,13,14]. Our reoperation rate was lower than the percentages reported in the literature, which varied from 2% to 3% [10,11,12,13,14].

Functional outcomes were significantly improved after the BPCP. OAB decreased in the majority of the subjects, while de novo urgency rate was very low. This data is similar to those reported in other surgical techniques [22,23].

A possible criticism to our technique is the potential increase of dyspareunia due to plication of the bilateral pubococcygeus muscle fibers. However, in our population, this surgical procedure did not significantly influence the pain domain of the FSFI, nor did it have a negative impact on female sexual function. Another potential criticism of our technique is the larger and deeper dissection between the bladder and endopelvic fascia when compared to standard anterior colporrhaphy. However, intraoperative adverse events were rare and easily managed.

## 5. Conclusions

Our surgical technique, the BPCP, was a safe and effective treatment to manage anterior vaginal wall defect and provided a robust native tissue support to standard anterior colporrhaphy.

## Figures and Tables

**Table 1:** Patients characteristics.

N° Pts.	147
POP-Q stage, % (*n*)	
2	49% (72/147)
3	51% (75/147)
Ba mean (SD)	1.2 (1.0)
Aa mean (SD)	1.4 (1.2)

POP-Q, Pelvic Organ Prolapse Quantification.

**Table 2:** Patient outcomes.

Follow-Up, Months Mean	82.4 Months (Range 24–120)	
**Objective success**		
POP-Q <2 stage, % (*n*)	89.8% (132/147)	
Aa mean (SD)	−2.4 (0.9)	
Ba mean (SD)	−2.1 (1.1)	
**Recurrences, % (*n*)**	**Pre-operative POP-Q stage**	**Follow-up POP-Q stage**
Tot 10.2% (15/147)		
11.1% (8/72)	2	2
8.0% (6/75)	3	2
1.3% (1/75)	3	3

POP-Q, Pelvic Organ Prolapse Quantification.

**Table 3:** Functional outcomes on OAB.

Patients Affected by OAB Symptoms		*p* Value
OAB before surgery	36.7% (54/147)	<0.05 *
OAB after surgery		
In overall population	14.3 (21/147)	
In OAB patients	38.9% (21/54)	
De Novo OAB	3.2% (3/93)	
OAB-screener		
Before surgery	23.1 (8.8)	<0.05 *
After surgery	13.1 (5.9)	

OAB, Overactive bladder; * T-Student test.

**Table 4:** Sexual function outcomes.

	PRE	POST	*p* Value
**FSFI**	Mean (SD)	Mean (SD)	
Total score	17.1 (4.1)	18.2 (4.5)	>0.05 *
Desire	2.5 (0.9)	3.2 (1.0)	>0.05 *
Arousal	2.3 (1.4)	2.9 (1.6)	>0.05 *
Lubrication	2.6 (1.6)	2.9 (1.9)	>0.05 *
Orgasm	2.5 (1.3)	2.7 (1.9)	>0.05 *
Satisfaction	2.2 (1.5)	3.1 (2.0)	<0.05 *
Pain	2.6 (1.6)	3.4 (2.1)	>0.05 *

FSFI, Female Sexual Function Index; * T-Student test.

**Table 5:** List of complications and Clavien–Dindo classification.

**Complications**		
Intraoperative bladder injury	2 (1.4%)	
Hematoma	3 (2.0%)	
Pain	2 (1.4%)	
**Clavien-Dindo Classification**		
Grade I	None	
Grade II	None	
Grade IIIa	1	Percutaneous drainage of hematoma
Grade IIIb	1	Surgical (trans vaginal) drainage of hematoma and blood transfusion
